# Lecture on the Results of the Surgical Treatment of Cancer

**Published:** 1865-06

**Authors:** T. Spencer Wells

**Affiliations:** Surgeon in ordinary to Her Majesty’s Household


### LECTURE ON THE RESULTS OF THE SURGICAL TREATMENT OF CANCER.

By T. SPENCER WELLS, F. R. C. S.,

Surgeon in ordinary to Her Majesty's Household.

*Gentlemen* :—Perhaps there is no question which more frequently arises in practice, or which is answered with so much doubt and hesitation, as the question, "Should a cancer be removed ?" At one time removal was the general rule of practice. Then the frequency of return after removal, and the revival of the humoral or chemical pathology, led to the rule to operate in exceptional cases. It was ascertained that by removal of a cancer in the early stage, we only removed one seat of deposit, left the blood unaltered, encouraged deposit in some other part, and so did more harm than good, often shortening life. Therefore it came to be a sort of rule of this school not to attempt to remove or destroy a cancerous tumour, unless it was either actually ulcerated, or growing so fast that the skin was about to give way, or the destruction of some vital part was threatened. It was urged that growths, having all the characters of cancer, occasionally disappeared, under the influence of hygienic or medical treatment—that other such growths have sometimes remained completely dormant for many years without injuring the health or shortening the life of the individual—and that it was absurd to say the disease was not cancerous in such cases because the patient recovered, or lived to old age unaffected by the local disease. But the most determined leaders of this school admitted that in cases where an open cancer gives great pain, and is wearing away the patient by bleeding or profuse discharge, this cancer should be removed, in the hope first of relieving suffering, and secondly, of prolonging, though not saving life. In some other cases where a cancer caused great mental anxiety to a patient, it was to be removed at her earnest entreaty, after fairly explaining the probability of a return of the disease. For many years these rules were followed in practice, and are so still by many excellent surgeons. But during the last four years a marked change has been taking place, and the professional mind is still in a state of transition. The transudation hypothesis is falling before the cellular theory. The belief that cancer is always a disease of the blood is severely shaken by the vigorous attacks of Virchow, and the positive and visible demonstration which he affords of the local origin of morbid growths, and the secondary contamination of neighboring parts and of the blood by extension and by absorption of the products of local disease. He has gone very far towards proving that the cells of the connective tissue (commonly known as cellular or areolar tissue) are the ovules of cancerous growths, as they are of all the new cellular productions. We are still ignorant of the nature of the peculiar change or irritation which, in the first instance, alters a connective tissue cell into a cancer cell; but when once this change has taken place, and the altered cells begin to grow and multiply by division, we have clearly a focus both of local and general contamination—local, by imbibition of the fluid formed by the diseased cells into the healthy cells of adjacent tissues; and generally by absorption or transudation of the morbid products through the lymphatics and veins. Thus, the harder the tumor, the less likely to spread; the softer, the more likely both to spread locally and to contaminate the blood. The direct and necessary practical deduction for the surgeon from these views is, that all malignant growths should be removed in their earliest possible stage—as soon, in fact, as their nature is ascertained; and to this rule, surgeons seem to be partially, though perhaps unconsciously, returning.

It requires the observation, and careful observation of many years, and of many independent observers, to settle such a point as this; but a great deal has been done to show, by comparing a large number of cases of cancer where removal has been practised with others where the patient has been left without operation, that the average duration of life in the patient operated on is decidedly lengthened. No trustworthy data exist for making such a comparison with cases in which the tumor was removed at a very early stage, but my own experience is leading me to adopt the rule deduced from the pathology of Virchow. I have had several cases since the publication of my pamphlet on "*Cancer Curers*," in which I have excised growths which microscopic examination has proved to be truly cancerous, and in which no return has yet been observed. The time is obviously too short to speak of them as permanent cases—indeed, it is almost certain that in nearly all, there will, sooner or later, be a return; but the result has satisfied me that the rule of practice most commonly followed may be advantageously modified in cases where the disease is in a very early stage. The operation at that stage is a very trifling one, is perfectly painless under chloroform; and so little necessity does there appear to be to keep patients to their rooms, that I have both in private practice and among hospital out-patients allowed them to come backwards and forwards from their homes. Supposing the general health to improve, and the cause which led to the first change in the connective tissue cells should not operate again, then *removal* really becomes *cure*.

If the teaching of Virchow, the greatest of living pathologists, be true—if the belief in the universal constitutional origin of cancerous tumors be unfounded—if the constitutional disease is, even in some few cases, secondary, the morbid condition of the blood originating in, and being kept up by a supply of hurtful ingredients from some diseased locality—how hopefully may both surgeon and patient look upon the treatment of cancer in its early stages, before the germs have spread into neighboring tissues, before they or any irritating fluid have been conveyed to a distance, before the healthy cells of neighboring parts have imbibed by endosmosis the specific or parenchymatous fluid, or this fluid has been carried by lymphatics or veins to distant parts. And, on the other hand, how fearful is the responsibility of the cancer curer who tampers with the life of the poor sufferer who has confided in his knowledge and skill! If he attempt to procure absorption of the tumor and succeed, he may produce the general contamination of the blood of which the growth itself was erroneously regarded simply as an "outward and visible sign." If he fail, he still gives time for the growth of germs, and for extension first locality, and then by absorption. If he employ caustics, he may be adopting the very practice of all others most likely to lead to a rapid and general development of cancer throughout the body, by hastening softening of the growth, setting up irritation in neighboring parts, and assisting the transmigration of cancer cells through the blood to distant parts of the body.

The fact that *removal* may really result in *cure*—that the disease may not return—should certainly encourage both patient and surgeon to think of removal in any case where the whole of the diseased part can be removed without risk or difficulty; but it would be very wrong to conceal from any patient the fact that this happy result is extremely rare, and that, except under very unusual circumstances, no more can be expected from removal than prolongation of life, and diminution of suffering.

That *life is prolonged* by removal is now satisfactorily established by statistics. The results of different statistical tables vary. But those most to be relied on tend to prove that the average duration of life in those operated on is certainly greater than in those in whom the disease is allowed to run its course without operation. In a valuable lecture, published in the *Medical Times and Gazette*, in 1862, Mr. Paget says: "In a recent tabulation of hospital and private cases, 85 cases operated on lived an average of 55.6 months, and 62 cases not operated on lived an average of 43 months. And such proportion as this will probably always be found."

Not only is life prolonged, but *suffering is lessened.* The amount of suffering, mental and bodily, of a patient during the ordinary course of cancer is very great. "Consider," says Mr. Paget, "the pain and anxiety—the pain likely to increase daily—the misery of waking every day to the consciousness of an incurable disease; the sometimes loathsomeness; the restlessness for cure—cure, such as there are *never wanting dishonest men to promise.*" By an operation painless under chloroform, and not followed by painful dressings, this constant, daily, weary pain is relieved for a time, and the patient is also restored to a state of mental comfort which is as great a gain as the relief from pain. In some cases, where there is no hope of prolonging life, the pain and suffering from an open cancer are so great that it is right to operate simply to obtain for the patient a comparatively painless death.

If, then, the oft-repeated objection to operation—that life is not lengthened but shortened—is satisfactorily answered; if we also dismiss as unproved, and certainly erroneous, the suggestion that when the disease returns it will be in a more aggravated form than if the disease had run its own course, the question arises, "Why not operate in every case?" And the answer is ready, "Because the operation is one attended with risk to life." In some cases this risk is very great, in others very small; but taking the averege of some hundreds of cases, Mr. Paget estimates that "a patient runs a risk of 1 in 10 of dying" from the operation. Others estimate the risk as low as 5 per cent., or 1 in 20.

In a consultation, however, upon any individual case, neither patient nor surgeon will be content with a mere statement of averages. It is seen at once, as from 1 in 20 to 1 in 10 die of the immediate effects of the operation, and the average life of those operated on is over 55 months, that some patients must live many years. And the questions which are asked are not: What are the *general* results of operation? How long do people *in general* live after it? How soon does the disease *generally* return after operation? but the individual case is pressed home: What risk would there be to *my* life? How long shall *I* be likely to live? How soon may *I* expect a return of the disease?

It is said, and said truly, that general conclusions can only be drawn from general average. Mr Paget says: "To deal with single cases is a sort of surgical gambling." The gamblers, however, are they who operate in nearly every case which comes before them and they who operate in none, both classes satisfying themselves by statistical tables and general averages, rather than by forming a careful estimate of the probable results of treatment in each case. Each case must be considered by the light of general experience, but it is no less in itself a subject for separate calculation. And in every case which comes before us we must be guided by certain general rules, the most important of which may be shortly summed up as follows:—

*Risk of Removal.*—The average mortality being from 5 to 10 per cent., in what cases is the risk above, and in what below the average? Supposing the patient to be placed under the most favorable circumstances possible to prevent the occurrence of erysipelas, pyæmia, any form of septiæmia, or sendary hemorrhage (the chief causes of death *soon* after operation) the probable liability of any patient to these dangers may be estimated by observing certain general and local conditions. The *general* conditions favorable to operation, or those which may fairly justify the hope that a patient will not die from its effects, are that she is not more than 60 years of age, not emaciated, is well-nourished, clear complexioned, temperate, courageous and even-minded, and free from other organic disease. The reverse conditions are seen in patients over 60 years of age—in over or under fed people—the emaciated, or the fat and plethoric—those of cachectic, leukæmic, or chloro-anæmic aspect—the dejected or hysterical—and they who, in addition to the cancer, have some organic disease of heart, lungs, liver or spleen. The favorable *local* conditions are that the skin over and near the tumor is soft and healthy, free from œdema, freely movable, and free from scattered nodules of cancer—that there are no diseased cervical glands, none, or few diseased auxillary glands—that the breast is small—that only one breast is diseased—and, if only one part of one be affected, that this is the lower part. The unfavorable local conditions are, the skin is brawny, the hair-follicles large and open, the skin over the tumor adherent, that there are scattered cancerous tubercles in the skin, that the supra-clavicular glands are swollen, that there are many diseased axillary glands, that the breasts are large, that both are diseased, or, if only part of one, that it is the upper part.

By bearing these things in mind it is not difficult so to select cases as to reduce the risk of removal to a very low average, and there can be no difficulty in deciding in any case whether the risk is greater or less than the average.

When part of one breast only is affected, the rule generally followed is to remove the whole breast; and this rule is a good one if the breast is small. But when only a small portion of a large breast is affected, it is much less dangerous and equally effectual to remove the diseased part and some of the surrounding healthy tissue, than to remove the whole breast. There is good reason to believe that the disease is as long in returning when only the diseased part has been removed, as when the whole breast is removed.

When it has been decided to remove cancer, the question will arise, "Should it be removed by the knife, or by caustic?" And the answer must be framed again upon full consideration of all the circumstances of each case, but guided by the following general rules:

1. There is no proof whatever that the return of cancer after removal by caustic is less rapid than after removal by the knife. On the contrary, it is probable that the return will be more rapid, (*a*) from the greater probability that some portion of the cancer will escape the action of the caustic than the dissection of a careful surgeon; (*b*) from the inflammation set up in the surrounding tissues and the irritation of neighboring glands.

2. Admitting that the immediate risk to life from the removal by caustics may be less than after removal by the knife —in other words, that pyæmia and hemorrhage are less likely to occur—we have abundant proof that the risk is very considerable, and that the intense and long-continued suffering does in itself lead to exhaustion and death.

3. It is possible that the attempt to remove a cancer by caustics may fail altogether, that the irritation set up in the diseased growth and in the parts surrounding it, or the extension of the sloughing to vital parts in the neighborhood, may destroy the patient before the cancer is destroyed.

These objections will generally lead the surgeon to advise the patient rather to submit to excision than to caustics. But in some cases, where the situation of the growth is such that the knife cannot be used safely, caustics are decidedly preferable, as they are also in cases where the growth is small, or of moderate size, and slow in its progress, while the unhealthy general condition of the patient would render the knife more than usually hazardous; for, as Mr. Paget has said, "the constitutional maladies that greatly increase the risk of cutting, do not, except very rarely, increase the risk of caustics." There are also some cases of open cancer in a very late stage of the disease, so adherent that the knife could not be safely used, where the diseased part may be completely destroyed by caustic, and a sound cicatrix may result. Sometimes it may be well to leave the choice to a patient, after representing to her fairly the advantages of the two methods. Some may be guided by the dread of the knife, others more by the dread of pain, and it is right to allow these fears their full weight.

The cases decidedly unfit for caustics are those of acute or rapidly-growing cancer, or those of large size, and those where there is an œdematous, brawny, or tuberculated or ulcerated condition of the surrounding skin, and those where the supra-clavicular or axillary glands are enlarged and indurated. And most of the conditions which on general grounds forbid the use of the knife also forbid the use of caustic.

Supposing it to be decided to destroy a cancerous tumor by caustic, all the evidence before us goes to prove the chloride of zinc to be the most effectual and safest yet employed; that it is a matter of great indifference whether it is employed as a paste or in solution; but that its action is considerably hastened by scoring through the slough, as Justamond did, down to the living tissues beneath, so that they are not protected by the slough from the action of the caustic. The scoring is not so necessary when the chloride is used in solution as when it is used as paste, after destroying the skin by nitric acid; and it is not at all necessary if one or more pairs of galvanic plates are used as the caustic. If a piece of zinc be placed on any raw surface, and a piece of silver near it, a silver wire connecting the two, the part covered by the zinc is destroyed very rapidly, and the slough formed is a very soft one, which is easily sponged away. I saw a lady in 1854, with Dr. Lawrence, of Connaught-square, suffering from cancer of the breast, and we decided, on consultation, to adopt this method, which Dr. Lawrence carried out most effectually. I should not be at all surprised to hear that the next great empiric who appears in London will profess to cure cancer by galvanism.

Occasionally it is advisable to apply the actual cautery instead of using caustic, and I have recently found the use of a small jet of lighted gas, by means of the instrument made by Mathieu for M. Nelaton, very efficacious. It is much more easily arranged than the galvanic cautery, is equally manageable, and has the great advantage of destroying parts without touching them; so that there is no fear of the eschar adhering to the metal, being removed as the metal is withdrawn, and bleeding thus being set up. In cancer of the upper jaw, or base of the skull, which project into the mouth, very great temporary relief is gained, even if only a partial destruction of the growth can be effected.

When removal cannot be recommended either by the knife or by caustics, it must be remembered that we can do a great deal more towards arresting, even curing cancer, than is generally believed—that our art is not nearly so powerless as charlatans assert. Growths with all the characters of cancer have occasionally, although very rarely, disappeared under the influence of remedies. Other such growths have remained completely dormant for many years, without affecting the health or shortening the life of the individual; and it is absurd to say that the disease was not cancerous in such cases because the patient recovered, or lived to old age unaffected by the local condition.—*Med. Times and Gazette*, Feb. 11, 1865.

